# Modeling Infection and Tropism of Human Parainfluenza Virus Type 3 in Ferrets

**DOI:** 10.1128/mbio.03831-21

**Published:** 2022-02-15

**Authors:** Laurine C. Rijsbergen, Katharina S. Schmitz, Lineke Begeman, Jennifer Drew-Bear, Lennert Gommers, Mart M. Lamers, Alexander L. Greninger, Bart L. Haagmans, Matteo Porotto, Rik L. de Swart, Anne Moscona, Rory D. de Vries

**Affiliations:** a Department of Viroscience, Erasmus MC, University Medical Centre, Rotterdam, the Netherlands; b Center for Host-Pathogen Interaction, Columbia University Vagelos College of Physicians and Surgeons, New York, New York, USA; c Department of Pediatrics, Columbia University Vagelos College of Physicians and Surgeons, New York, New York, USA; d Department of Laboratory Medicine and Pathology, University of Washington, Seattle, Washington, USA; e Vaccine and Infectious Disease Division, Fred Hutchinson Cancer Research Center, Seattle, Washington, USA; f Department of Experimental Medicine, University of Campania “Luigi Vanvitelli,” Caserta, Italy; g Department of Physiology and Cellular Biophysics, Columbia University Vagelos College of Physicians and Surgeons, New York, New York, USA; h Department of Microbiology and Immunology, Columbia University Vagelos College of Physicians and Surgeons, New York, New York, USA; Duke University School of Medicine

**Keywords:** animal models, parainfluenza virus, viral pathogenesis

## Abstract

Human parainfluenza virus type 3 (HPIV-3) is a significant cause of lower respiratory tract infections, with the most severe disease in young infants, immunocompromised individuals, and the elderly. HPIV-3 infections are currently untreatable with licensed therapeutics, and prophylactic and therapeutic options are needed for patients at risk. To complement existing human airway models of HPIV-3 infection and develop an animal model to assess novel intervention strategies, we evaluated infection and transmission of HPIV-3 in ferrets. A well-characterized human clinical isolate (CI) of HPIV-3 engineered to express enhanced green fluorescent protein (rHPIV-3 CI-1-EGFP) was passaged on primary human airway epithelial cells (HAE) or airway organoids (AO) to avoid tissue culture adaptations. rHPIV3 CI-1-EGFP infection was assessed *in vitro* in ferret AO and in ferrets *in vivo*. Undifferentiated and differentiated ferret AO cultures supported rHPIV-3 CI-1-EGFP replication, but the ferret primary airway cells from AO were less susceptible and permissive than HAE. *In vivo* rHPIV-3 CI-1-EGFP replicated in the upper and lower airways of ferrets and targeted respiratory epithelial cells, olfactory epithelial cells, type I pneumocytes, and type II pneumocytes. The infection efficiently induced specific antibody responses. Taken together, ferrets are naturally susceptible to HPIV-3 infection; however, limited replication was observed that led to neither overt clinical signs nor ferret-to-ferret transmission. However, in combination with ferret AO, the ferret model of HPIV-3 infection, tissue tropism, and neutralizing antibodies complements human *ex vivo* lung models and can be used as a platform for prevention and treatment studies for this important respiratory pathogen.

## INTRODUCTION

Respiratory viruses affect millions of children globally each year, are an important cause of mortality in the elderly, and are a growing problem in the immunocompromised (1–4). However, for most of these viruses no specific vaccines or treatments are available. Human parainfluenza virus (HPIVs) types 1, 2, 3, and 4, human metapneumovirus (5), rhinovirus (6), and human respiratory syncytial virus (HRSV) cause the majority of childhood lower respiratory tract diseases (1, 7–10). The most common manifestations of acute respiratory infections in young infants (croup, bronchiolitis, and pneumonia) are caused by HPIV-3 and HRSV, with human metapneumovirus being important in older infants (11). Vaccines for HPIV-3 are under investigation; however, these vaccines are not yet close to implementation, and the most vulnerable populations are unlikely to be protected by any vaccine (12–23). While the use of corticosteroids has decreased hospitalizations for HPIV-1-associated croup (24), HPIV-2, HPIV-3, and HPIV-4 infections lack licensed therapeutics (9, 10, 14, 25–27). HPIV-3 is responsible for more hospitalizations than HPIV-1 and -2 combined (8, 28, 29). HPIV-3 transmission occurs via the respiratory route, and inpatient nosocomial outbreaks are common, causing considerable morbidity and mortality in high-risk groups (2, 30–35). Academic hospitals have seen a shift in the profile of HPIV-3 patients, resulting in an increased number of immunocompromised patients with severe comorbidities. This all points to the need to accurately model these viral infections and address prevention and treatment.

The parainfluenza virus types 1 and 3 belong to the *Respirovirus* genus within the *Paramyxoviridae* family, while HPIV-2 belongs to the *Rubulavirus* genus. HPIVs contain single-stranded negative-sense RNA and a lipoprotein envelope derived from infected cells (36). HPIV enters cells by fusing directly with the cell membrane in a process mediated by the two surface glycoprotein spikes, the receptor-binding protein hemagglutinin-neuraminidase (HN) and the fusion (F) protein. Ciliated epithelial cells in the respiratory tract are the main site of viral entry. Initial virus replication occurs in the upper respiratory tract (URT; nasal cavity and nasopharynx), after which the virus can disseminate to the lower respiratory tract (LRT; bronchi and bronchioles). Viral loads peak between 2 and 5 days after infection according to data from both *in vitro* and *in vivo* studies (37, 38). Despite the development of neutralizing antibodies in response to HPIV-3 infection in immunocompetent individuals, reinfections are a hallmark of HPIV-3 in children (3, 37).

Mechanisms that affect HPIV-3–host cell interplay are finely tuned to the host environment, and clinical isolates differ significantly from laboratory viruses in terms of receptor interaction and viral fusion properties that govern entry and fitness *in vivo* (39–42). Growing HPIV-3 in standard cell culture leads to immediate adaptations in the fusion/entry complex and the characteristics that confer viral fitness. However, in human airway epithelial (HAE) cells and human lung organoid models (airway organoids [AO]), clinical strains of HPIV-3 grow and spread without adaptation (43). Such human airway models permit the study of HPIV-3 tropism and pathogenesis in the human respiratory epithelium in the absence of an adaptive immune response (39–45). In *ex vivo* human lung tissue, HPIV-3 primarily infects ciliated epithelial cells and type II pneumocytes (43, 46). Several hallmarks of HPIV-3-associated disease were demonstrated in AO, including viral shedding without cytopathology or epithelial cell sloughing (43). In HAE, infection occurs at the apical surface and HPIV-3 replicates in the absence of gross cytopathology (43, 46), in both cases similar to observations in humans infected with HPIV-3.

A number of animal species have been evaluated as *in vivo* models for HPIV-3 infection: cotton rats, hamsters, guinea pigs, nonhuman primates, mice, and ferrets (47–49). In most studies, animals were inoculated intranasally (i.n.) with 10^5^ to 10^6^ 50% tissue culture infectious doses (TCID_50_), but limited information is available on the passage history of the virus used in these studies. Based on recent findings regarding rapid viral adaptation, the virus stocks were likely to be tissue culture adapted. Few animals developed clinical signs, with the exception of newborn ferrets, in which HPIV-3 infection was lethal, and African Green monkeys, which developed nasal discharge. The peak of infection was, on average, between 2 and 5 days postinoculation (dpi), similar to the peak of shedding in humans (38, 50–56). Animal-to-animal transmission was exclusively demonstrated in guinea pigs (57). Limited histological data about HPIV-3 infection exists for either humans or animals; infection of the LRT was demonstrated in cotton rats and hamsters, but involvement of the URT has not been described. Typical signs of viral respiratory disease were observed in cotton rats, including peribronchiolitis, alveolar wall thickening, lymphocyte influx, and mild epithelial cell degeneration (38, 54, 56). HPIV-3 infected mainly ciliated epithelial cells in the cotton rat bronchi and bronchioles but was also detected in type II pneumocytes and alveolar macrophages (38, 56). Cotton rats, hamsters, and guinea pigs usually recovered from HPIV-3 infection and developed robust antibody responses (38, 50, 54, 56).

To develop an animal model that is fit for the purpose of studying HPIV-3 infection, tropism, transmission, and virus-specific immune responses to test intervention strategies, we combined a recombinant HPIV-3, based on a well-characterized clinical isolate (CI) (41) and engineered to express enhanced green fluorescent protein (EGFP), with the ferret model. Ferrets are naturally susceptible to human respiratory viruses, including influenza virus, HRSV, and severe acute respiratory syndrome coronavirus 2 (SARS-CoV-2) (58, 59). We first established ferret AO, characterized rHPIV-3 CI-1-EGFP replication *in vitro*, and directly compared HPIV-3 replication kinetics to those of human AO. Next, we studied infection, tropism, and transmission of HPIV-3 in ferrets after i.n. and intratracheal (i.t.) inoculation with different concentrations of virus. This study establishes an infection model for this important pediatric pathogen and lays the groundwork for further prevention and treatment studies in a model that complements human *ex vivo* lung models and existing HPIV-3 animal models (40–43).

## RESULTS

### Ferret AO cultures support rHPIV-3 CI-1-EGFP replication.

**FIG 1:**
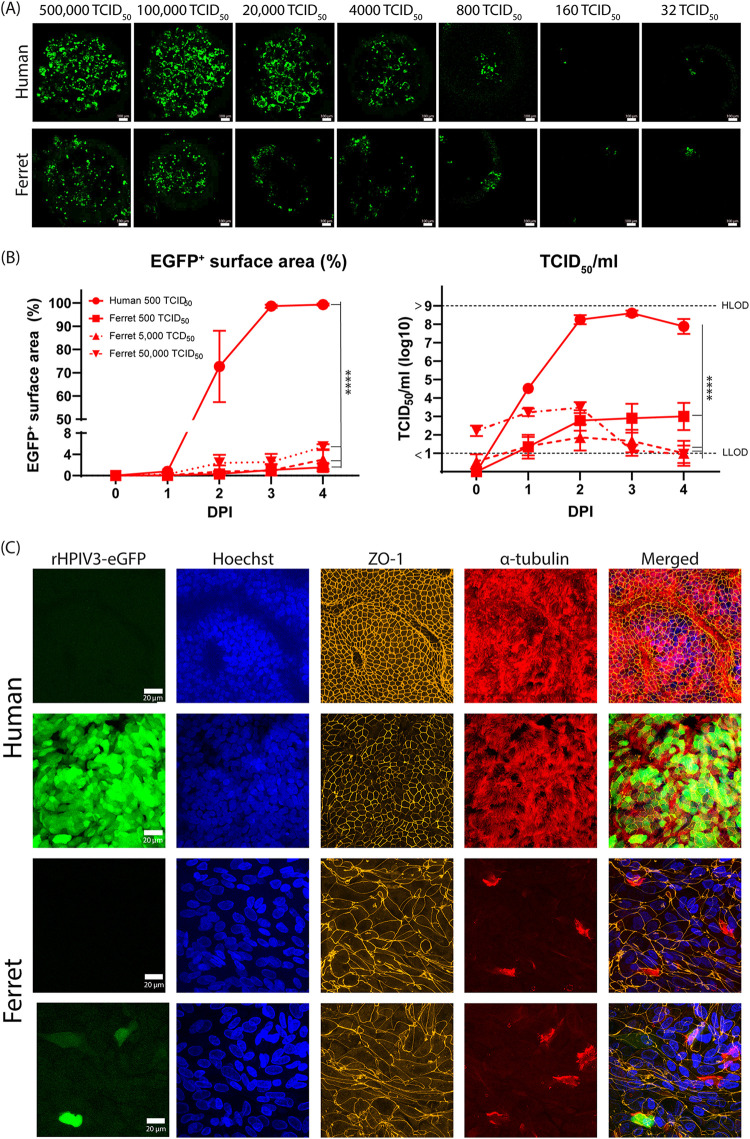
rHPIV-3 CI-1-EGFP replication kinetics in ferret AO and AO at ALI. (A) Undifferentiated ferret and human AO were inoculated with rHPIV-3 CI-1-EGFP at the indicated dose and monitored for 3 days. Representative images at 3 dpi are shown. (B) Ferret and human AO at ALI were inoculated with 500, 5,000, or 50,000 TCID_50_ rHPIV-3 CI-1-EGFP and monitored until 4 dpi. The EGFP^+^ surface area was determined by tile scans based on fluorescence, and viral titers were determined by endpoint titrations of apical washes (TCID_50_/mL). (C) Ferret and human AO at ALI were fixed in 4% paraformaldehyde (PFA) at 4 dpi and used for indirect immunofluorescence using antibodies against zona occludens-1 (tight junctions, orange), acetylated α-tubulin (cilia, red), and Hoechst (nuclei, blue). Representative images are shown. Two independent experiments are shown, and all experiments were performed in triplicate. Differences between the growth curves were statistically analyzed by two-way analysis of variance (*, *P* = 0.05; **, *P* = 0.01; ***, *P* = 0.001; ****, *P* < 0.0001). Means and individual replicates are shown.

To determine whether primary respiratory epithelial cells from ferrets are susceptible and permissive to HPIV-3 infection, we inoculated undifferentiated ferret AO with rHPIV-3 CI-1-EGFP. The virus infected undifferentiated ferret AO at all tested concentrations, although infection was less efficient than that in undifferentiated AO of human origin (Fig. 1A). We further assessed replication kinetics in differentiated ferret epithelial cell cultures from AO at air-liquid interface (ALI) using three doses of rHPIV-3 CI-1-EGFP (500, 5,000, or 50,000 TCID_50_). Although ferret AO at ALI were infected, as assessed by fluorescent surface area and infectious virus titer, infection was less efficient than that of human AO at ALI (Fig. 1B). To study the tropism of HPIV-3 in ferret AO at ALI, we counterstained filters for tight junctions (ZO-1) and cilia (acetylated α-tubulin). We observed that rHPIV-3 CI-1-EGFP mainly infected ciliated cells in human AO at ALI but both ciliated and nonciliated cells in ferret AO at ALI (Fig. 1C). However, compared to human AO at ALI, the ferret AO at ALI contained fewer ciliated cells; only 1 to 2 cell layers (compared to 3 to 5 cell layers in human AO at ALI) were observed, and the morphology of the cells was less cuboidal. Combined, while ferret primary airway cells were susceptible and permissive to rHPIV-3 CI-1-EGFP, there was measurably less infection and replication than for human primary airway cells.

### rHPIV-3 CI-1-EGFP replicates in ferrets but does not lead to clinical signs or transmission.

**FIG 2:**
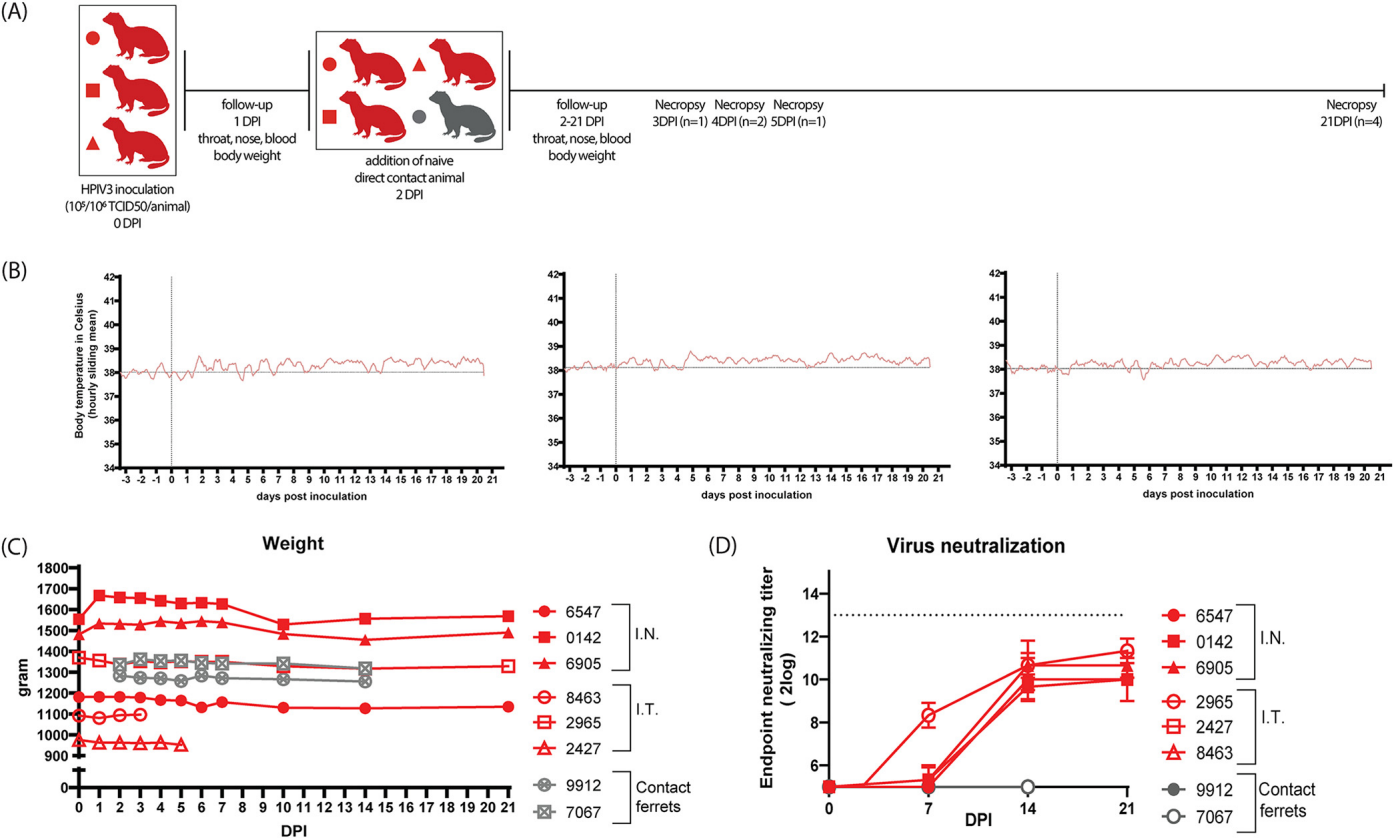
Design of *in vivo* experiment, clinical signs, and absence of direct contact transmission after rHPIV-3 CI-1-EGFP inoculation. (A) Design of the ferret experiment. The red symbols represent experimentally inoculated ferrets, either intranasal (i.n.) or intratracheal (i.t.), and the contact ferrets are represented by the gray symbols. (B) Body temperature of 3 ferrets that were inoculated intranasally with 10^5^ TCID_50_ rHPIV-3 CI-1-EGFP. The hourly sliding mean is shown, and the vertical line represents the day of inoculation. (C) Body weights, in grams, of experimentally inoculated ferrets during the course of the experiment (red lines and symbols) and contact ferrets (gray lines and symbols). (D) Endpoint neutralizing titers measured on Vero cells of both the experimentally inoculated and contact ferrets following rHPIV-3 CI-1-EGFP inoculation (bottom horizontal line represents the lower limit of detection, and upper horizontal line represents the upper limit of detection).

After confirming that undifferentiated and differentiated primary ferret respiratory epithelial cells are susceptible and permissive for HPIV-3 infection, we studied clinical signs, infection, tropism, transmission, and virus-specific antibody responses in ferrets *in vivo*. After i.n. or i.t. inoculation, ferrets did not develop clinical signs such as fever or weight loss (Fig. 2B and C). Furthermore, no direct-contact transmission was observed from inoculated to naive recipient ferrets, assessed by absence of viral genomes in throat and nose swabs and absence of neutralizing antibodies (Fig. 2D and data not shown). In the absence of clinical signs and transmission, all inoculated ferrets developed neutralizing antibody responses at 14 dpi, evidence of productive HPIV-3 replication (Fig. 2D).

**FIG 3:**
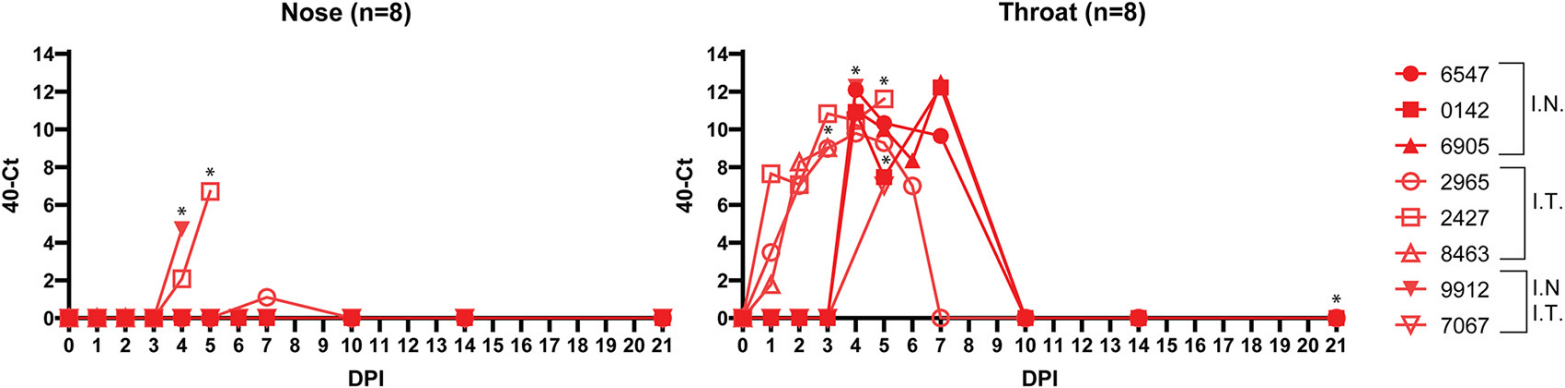
rHPIV-3 CI-1-EGFP replication kinetics in nose and throat swabs. Viral loads were detected in nose (left) and throat (right) swabs by RT-qPCR following i.n. and/or i.t. inoculation of ferrets. Asterisks indicate the date of euthanization of each animal. *y* axis = 40 minus the cycle threshold (40-Ct).

We monitored viral loads in throat and nose swabs obtained from 8 ferrets at the indicated time points postinoculation, *N* = 3 i.n. inoculated and *N* = 3 i.t. inoculated, and because the *N *= 2 direct-contact ferrets were not infected with HPIV-3 by transmission, we experimentally inoculated these naive animals i.n. and i.t. Viral RNA was detected in nose swabs collected from three animals between 4 and 7 dpi for all three inoculation methods (Fig. 3). In throat swabs we detected viral RNA in all eight animals and could detect virus as early as 1 dpi (in 3 animals that were inoculated i.t.), with the peak of infection at 4 to 8 dpi. We additionally obtained bronchoalveolar lavage (BAL) samples from 4 ferrets sacrificed around the hypothesized peak of infection and found HPIV-3-infected cells in these samples by flow cytometry (data not shown). These cells were productively infected, as virus could be isolated on human AO at ALI (data not shown). No EGFP^+^ cells were detected in the blood (measured in all 8 animals), indicating the absence of viremia. These results combined indicate that rHPIV-3 CI-1-EGFP replicates in the upper and lower respiratory tract of ferrets (with more virus detected in the lower respiratory tract), but replication does not result in overt disease or direct-contact transmission to naive cage mates.

### Cells in the URT and LRT of ferrets are targeted by rHPIV-3 CI-1-EGFP.

**FIG 4:**
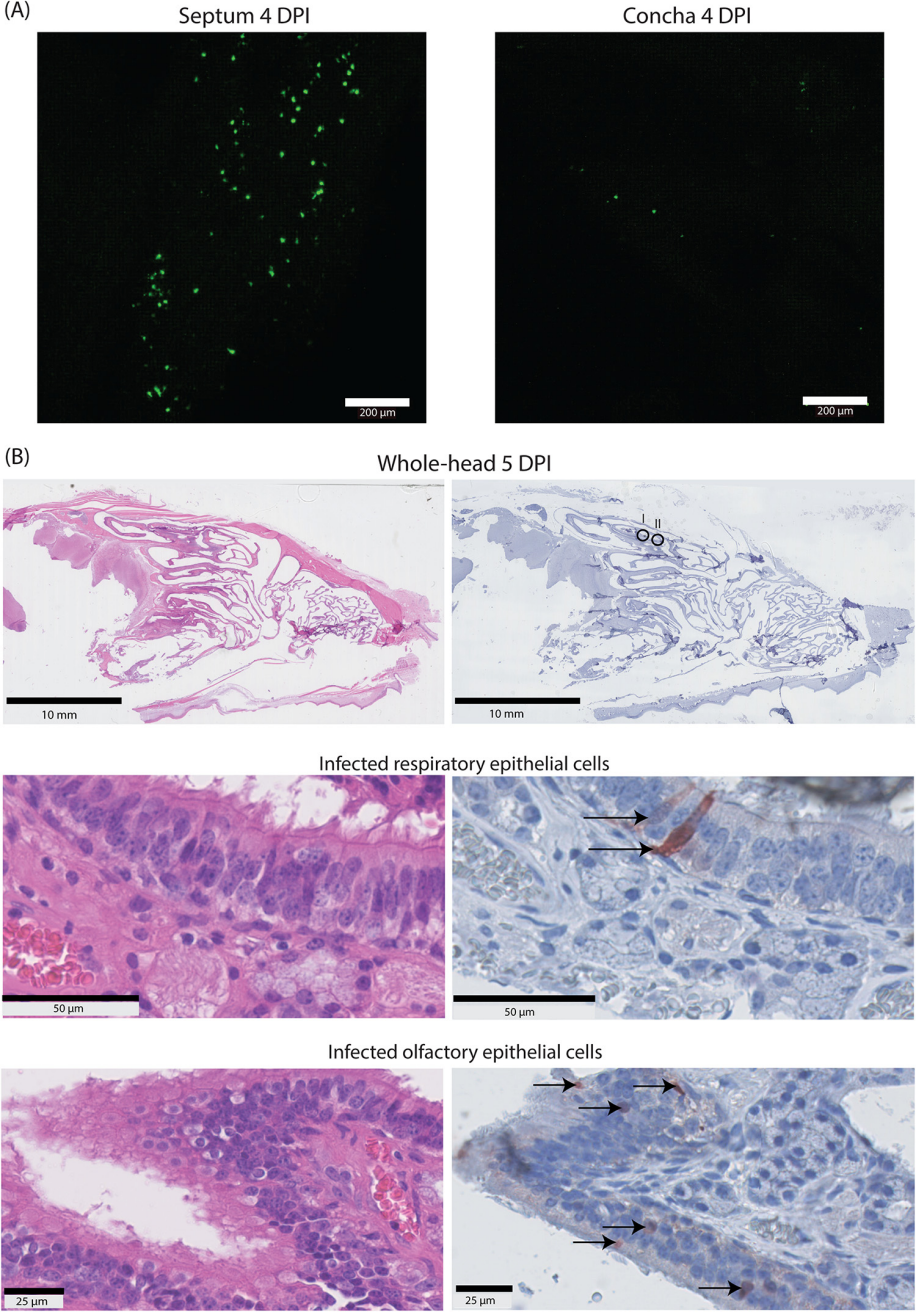
rHPIV-3 CI-1-EGFP dissemination and tropism in the upper respiratory tract of ferrets. (A) The nasal septum (4 dpi) and nasal concha (4 dpi) of rHPIV-3 CI-1-EGFP-inoculated ferrets were directly screened for fluorescence by confocal microscopy after necropsy, and representative images are shown. (B) Representative H&E and IHC images of a formalin-fixed whole head of a ferret inoculated with rHPIV-3 CI-1-EGFP (5 dpi). The whole heads are depicted in the top panels, while two areas with EGFP^+^ cells are shown enlarged in the bottom panels. Arrows in the enlargements indicate infected cells, and the EGFP^+^ cells are visible by homogenous to fine granular brown cytoplasmic staining.

To study the presence of rHPIV-3 CI-1-EGFP-infected cells in the URT, we screened the nasal septum and nasal concha obtained at necropsy by confocal microscopy. We detected single EGFP^+^ cells in the nasal concha (4 out of 4 ferrets sacrificed at the peak of infection, both i.n. and i.t. inoculation) and nasal septum (2 out of 4 ferrets, only i.n. inoculation) (representative images are in Fig. 4A). To further phenotype HPIV-3-infected cells in the nasal cavity and their potential association with lesions, we prepared tissue sections for histological analysis of the ferrets’ heads; this method completely preserved the architecture of the nasal concha (Fig. 4B). Similar to the concha obtained during necropsy, we detected EGFP^+^ cells in all animals sacrificed at the likely peak of infection (*n* = 4). In this analysis, both based on anatomical location and phenotype, we determined that both ciliated respiratory epithelial cells and olfactory cells were infected, and infected cells were predominantly located in the distal part of the nose. There were no associated histological lesions present in the areas with EGFP^+^ cells.

**FIG 5:**
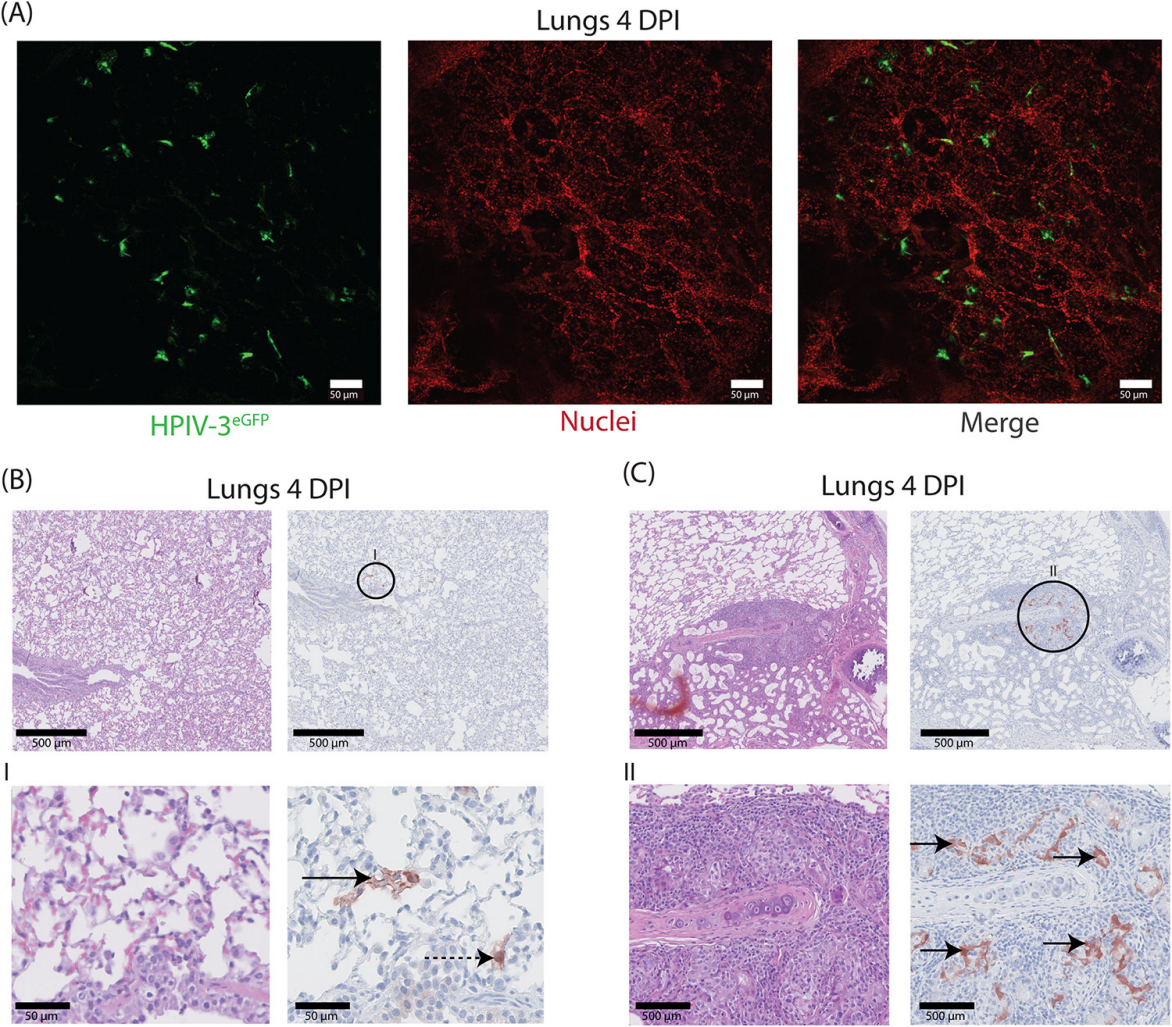
rHPIV-3 CI-1-EGFP dissemination and tropism in the lower respiratory tract of ferrets. (A) Lung slices from agarose-inflated lungs of rHPIV-3 CI-1-EGFP-infected ferrets (4 dpi) were fixed in 2% PFA and Hoechst stained for nuclei before imaging on a confocal microscope. Representative images are shown. (B and C) Representative hematoxylin and eosin and IHC images of formalin-inflated and formalin-fixed lungs from inoculated ferrets (4 dpi). The insets show areas with EGFP^+^ cells that are enlarged in the panels below, and rHPIV-3 CI-1-EGFP-infected cells are indicated by the arrows. (B) Infected type I (boldface arrow) and type II pneumocytes (dashed arrow). (C) Examples of rHPIV-3 CI-1-EGFP-infected bronchial glandular epithelial cells.

To study the presence of HPIV-3-infected cells in the LRT, we screened trachea rings and agarose-inflated lung slices collected from infected ferrets sacrificed at the peak of infection for EGFP. Although we rarely detected EGFP^+^ cells in the trachea of either i.n.- or i.t.-inoculated animals, we observed numerous clusters of fluorescent cells in the lungs of all animals. Studying both direct fluorescence in agarose-inflated lung slices and histology in formalin inflated lungs, we concluded that EGFP^+^ cells had a phenotype indicative of primarily type I and type II pneumocytes throughout the lungs without histological abnormalities (Fig. 5A and B). Both cell types contained a homogenously dispersed cytoplasmic brown staining. In one animal, dosed i.t. with 10^6^ TCID_50_ and sacrificed at 5 dpi, we additionally observed foci of EGFP^+^ cells in the peribronchial glands (Fig. 5C). EGFP^+^ cells showed necroses, and periglandular tissue showed infiltration of lymphocytes, plasma cells, and a few macrophages and neutrophils. Finally, we also discerned occasional EGFP^+^ alveolar macrophages in the lungs, although it is difficult to distinguish between direct infection or clearing of infected cells in these macrophages (data not shown).

## DISCUSSION

Comprehensive animal models for HPIV-3 that can be used to study hypotheses about infection, tropism, virus-specific immunity, transmission, prevention, and treatment of this prevalent pathogen in infants are needed to complement studies in human *ex vivo* models. Since ferrets are known to be naturally susceptible to most human respiratory viruses and HPIV-3 is known to rapidly adapt to cell culture, we modeled the course of infection with a validated clinical isolate-based HPIV-3 in ferrets and show that this offers a potentially useful approach for evaluating viral replication and development of antibody responses *in vivo* (58–60). We show that a clinical isolate-based HPIV-3 replicated in ferret AO at ALI and caused productive infection of ferrets *in vivo* with replication in both the URT and LRT. Respiratory epithelial cells, olfactory cells, and type I and type II pneumocytes were targeted. The productive infection, however, did not lead to overt disease or ferret-to-ferret transmission, but infected ferrets developed high neutralizing antibody titers.

To support *in vivo* ferret studies, we were the first to establish ferret AO and differentiate these at ALI. Though useful, these AO can be further optimized to perform combined *in vitro* and *in vivo* studies about HPIV-3 infectivity and to assess intervention strategies. The finding that primary ferret respiratory epithelial cells are less susceptible and permissive for rHPIV-3 CI-1-EGFP infection and replication than human respiratory epithelial cells is in line with our understanding of HPIV3’s fine specificity for specific host environments (39–42, 44, 45). While neonatal ferrets had been shown to be infectible by HPIV-3 that was almost certainly laboratory adapted (51, 52), we now show that clinical, nonadapted HPIV-3 strains infect ferrets *in vivo*. Future studies will determine whether viral evolution occurs toward adaptation to ferret cells and will identify the responsible genetic and functional changes. It will be of interest to note whether adaptation to ferret AO correlates with a concomitant decrease in growth in human cells and to identify the responsible changes. Ferret airway-adapted rHPIV-3 based on rHPIV-3 CI-1-EGFP may offer an alternative for use in this model, with the disadvantage of being less representative of the human clinical strain.

Combining the data from the different experiments, the peak of replication in ferrets as determined by viral genomes detected in the throat occurred between 4 and 8 dpi, which reflects replication in humans and other animal models (37, 38, 50, 53, 55, 56). We rarely detected viral RNA in the nose, likely due to a combination of minimal viral replication in the URT, as confirmed by immunohistochemistry (IHC), and sampling difficulties of the nasal cavity of ferrets. Our findings differ somewhat from those in ferrets infected with influenza virus, where the nasopharynx was a major site of viral replication, possibly due to the predominance of specific sialic acid-containing receptors (61), a question that will be addressed in future work. We did not observe weight loss or fever in HPIV-3-infected ferrets, in agreement with observations in cotton rats (38, 50–54, 56); only in neonatal ferrets and African green monkeys have clinical signs and/or lethality been observed (55). The age of ferrets may affect components of the response, and older ferrets may be important in the future for studying respiratory viral pathogenesis; for example, for SARS-CoV-2, more viral replication and viral shedding was seen in older ferrets than young ferrets (62). The absence of overt clinical signs in our model reflects HPIV-3 infection in humans to a certain extent, where severe disease is almost exclusively observed in young infants, immunocompromised, or the elderly, likely reflecting the presence or absence of neutralizing antibodies. The lack of direct-contact HPIV-3 transmission between ferrets is a disadvantage of the model, and we hypothesize this is due to the limited virus replication in the URT combined with the lack of clinical signs and therefore a lack of behaviors conducive to spreading infection (cough, coryza, etc). Transmission of HPIV-3 between animals has not been described, with the possible exception of a zoonotic HPIV-3 infection in guinea pig breeding units where HPIV-3-positive animals transmitted the virus to sentinel animals; in that case, the animals remained asymptomatic and proof of transmission was detected by serological testing (57). Inoculated ferrets similarly had high levels of neutralizing antibodies.

In humans, HPIV-3 initially replicates in the URT and subsequently disseminates to the LRT (37). Studies of human tissue have focused on the LRT and detected HPIV-3 mainly in ciliated epithelial cells of the bronchi and bronchioles, and occasionally in type II pneumocytes and alveolar macrophages (38, 43, 46, 56); pathological or histological evidence from infected children or adults is lacking. In this study, we examined both the URT and LRT and identified infected ciliated respiratory epithelial cells in the nasal concha but also observed infected olfactory epithelial cells. This is not unique to HPIV-3; other respiratory viruses, including SARS-CoV-2, influenza virus, and HRSV, also infect the olfactory cells (63–67). However, HPIV-3 infection in the URT of ferrets did not produce inflammation or cytopathology, as observed with other respiratory viruses (68). Surprisingly, in contrast to previous studies in cotton rats (38, 54, 56), we did not find infected cells at the apical side of the bronchi and bronchioles in the LRT. However, we observed that HPIV-3 infects type I and type II pneumocytes in the ferrets without cytopathology.

The ferret model we present here helps to set the platform for development of both antiviral therapeutics and vaccines. Although we did not detect ferret-to-ferret transmission, the model is an accurate reflection of HPIV-3 infections in humans: using a non-cell culture-adapted virus, we (i) observed limited clinical signs in immunocompetent animals, (ii) demonstrated similar tropism for ciliated epithelial cells as in humans with the potential to spread to the LRT, and (iii) detected a strong virus-specific immune response. Future development of the model will include methods for assessing lung function in these ferrets; airways resistance, airway hyperresponsiveness, and lung compliance among other assessments will complement the model’s value if we can detect subtle changes in lung function, even in the context of a less permissive host. The studies presented here lay an important foundation for further refinement of the model using immunocompromised ferrets, which was useful for the study of influenza virus and HRSV infections (60, 69, 70). In immunocompromised ferrets, the impact of interventions on HPIV-3 viral load, transmission, viral shedding, respiratory disease, and even the development of antiviral resistance can be further evaluated (70, 71). Additionally, there are currently several live-attenuated vaccines in development for HPIV-3 (13–22, 26, 72, 73), and immunogenicity and protective efficacy could be further evaluated in both immunocompetent and immunocompromised ferrets.

## MATERIALS AND METHODS

### Ethics statement.

Influenza virus, SARS-CoV-2, and Aleutian disease virus seronegative male ferrets (Mustela putorius furo) were obtained from a commercial breeder (Triple F Farms, PA). Animals were housed and experiments were performed in compliance with the Dutch legislation for protection of animals used for scientific purposes (2014; implementing EU Directive 2010/63). Research was conducted under a project license from the Dutch competent authority (license number AVD10100202011006), and the study protocol was approved by the institutional Animal Welfare Body (Erasmus MC permit number 20–11006-01). Animal welfare was monitored on a daily basis.

### Viruses and cells.

The generation of the rHPIV-3 clinical isolate 1 (CI-1)-EGFP (rHPIV-3 CI-1-EGFP) was previously described (40, 41). Virus stocks were grown on either HAE at ALI or AO at ALI by inoculating cells with 500 to 5,000 TCID_50_ and harvesting in Dulbecco’s phosphate-buffered saline (DPBS) supplemented with Ca and Mg (0.9 mM MgCl_2_ and 0.49 mM CaCl_2_) at 2 to 4 dpi. Viral stocks were titrated on Vero cells, and the TCID_50_/mL was calculated according to the Reed and Muench method (74).

### Airway organoid cultures.

Human AO cultures were generated based on published protocols (43, 63, 75–77). Ferret airway organoids were generated with minor adaptations to these protocols. Lung tissue obtained from uninfected ferrets euthanized in the course of other studies was used to obtain ferret lung stem cells from the distal airways. Undifferentiated human or ferret AOs were cultured in Matrigel (Corning) droplets (∼30 μL) with 250 μL AO medium and split every 10 to 14 days. To obtain differentiated organoid-derived cultures at ALI, organoids were disrupted into a single-cell suspension with TrypLE Express and seeded on Transwell membranes (Corning) coated with rat tail collagen type I (Fisher Scientific) in AO medium and complete base medium (CBM; PneumaCult-ALI; Stemcell Technologies) at a 1:1 ratio. When the monolayer was confluent (2 to 4 days), the cultures were placed on ALI in CBM. Cultures were differentiated for 4 to 6 weeks for human cultures and 2 to 4 weeks for ferret cultures with fresh medium every 5 days. Differentiation was confirmed by visualization of cilia and/or antibody staining for tight junctions (zona occludens-1) and cilia (acetylated α-tubulin).

### HPIV-3 growth kinetics in AO.

Infection of undifferentiated ferret and human AO was performed by inoculation with different inocula (500,000 to 32 TCID_50_ in 120 μl) of rHPIV-3 CI-1-EGFP without washing, and growth kinetics were assessed by fluorescence microscopy. Growth kinetics of rHPIV-3 CI-1-EGFP in differentiated human and ferret AO at ALI were assessed as follows. Cultures were washed apically with DPBS supplemented with Ca and Mg and inoculated apically with 500, 5,000, or 50,000 TCID_50_ rHPIV-3 CI-1-EGFP per filter. AO at ALI were submerged in the inoculum for 1 to 2 h at 37°C and 5% (vol/vol) CO_2_. After inoculation, cultures were washed twice with DPBS supplemented with Ca and Mg. To study replication kinetics, supernatant fluid from the apical side was collected by adding 200 μL DPBS supplemented with Ca and Mg, incubating for 10 min at 37°C and 5% (vol/vol) CO_2_, harvesting, and freezing at −80°C. The titer of virus in supernatant fluid was subsequently determined on Vero cells. The percentage of EGFP^+^ surface area was determined by imaging complete filters on an Amersham Typhoon and calculating the EGFP^+^ surface area using Fiji (78).

### Infection and transmission experiments in ferrets.

*In vivo* experiments were performed using ferrets that had no detectable HPIV-3-specific neutralizing antibodies (Fig. 2A). In the first experiment, three ferrets were inoculated i.n. with 1 × 10^5^ TCID_50_ rHPIV-3 CI-1-EGFP in 450 μl (225 μl instilled dropwise in each nostril) and housed together in a negatively pressurized HEPA-filtered animal biosafety level 3 (ABSL-3) isolator. In the second experiment, three ferrets were inoculated i.t. (3.5 mL via tracheal intubation) with 1 × 10^6^ TCID_50_ rHPIV-3 CI-1-EGFP and housed together in a negatively pressurized HEPA-filtered ABSL-3 isolator. Inoculation was considered the start of the experiment (0 dpi). In both experiments, at 2 dpi, a contact ferret was placed in the same isolator to study direct-contact transmission. Body weights were monitored, clinical scoring was performed, nasal and throat swabs were obtained daily from 0 to 7 dpi and at 10, 14, and 21 dpi, and blood samples were obtained at 0, 3, 5, 7, 14, and 21 dpi. Swabs were stored at −80°C in virus transport medium (minimum essential medium Eagle with Hank's balanced salt solution [Lonza], 5 g/liter lactalbumin enzymatic hydrolysate, 10% glycerol [Sigma-Aldrich], 200 U/mL penicillin, 200 mg/mL streptomycin, 100 U/mL polymyxin B sulfate [Sigma-Aldrich], and 250 mg/mL gentamicin [Life Technologies]) and used for reverse transcription-quantitative PCR (RT-qPCR) to determine viral loads. Blood was collected in serum-separating tubes, and the serum was processed, heat inactivated, stored at −20°C, and used to measure neutralizing antibodies against rHPIV-3 CI-1-EGFP. Whole white blood cells were run on a flow cytometer (FACSLyric; BD) to count EGFP^+^ cells. Since we could not detect HPIV-3 transmission to the contact animal after 12 days of cohousing (14 dpi, absence of transmission demonstrated by negative RT-qPCR and no seroconversion), contact animals were inoculated with 1 × 10^6^ TCID_50_ i.n. (225 μl per nostril) and i.t. (3.5 mL). Ferrets were euthanized at 3 dpi (*n* = 1), 4 dpi (*n* = 2), 5 dpi (*n* = 1), or 21 dpi (*n* = 4) via exsanguination following anesthesia. In 4 ferrets that were inoculated i.n., a temperature probe was implanted intraperitoneally to measure body temperature over the course of the experiment. All animal handling was performed under anesthesia using ketamine and medetomidine (100 mg/kg of body weight and 10 mg/kg, respectively).

At necropsy the respiratory tract was sampled. We obtained BAL fluid as well as trachea, lung, TB-LN tissues, and whole heads for further analysis. The lungs were either inflated with agarose (4 % [wt/vol] low-melting-point agarose) to prepare lung slices to screen for EGFP^+^ cells and perform counter staining with Hoechst (1:10.000) (79) or with formalin to screen for EGFP^+^ cells as well as lesions with light microscopy. The nasal concha, nasal septum, and tracheal rings were directly screened for fluorescence on a confocal laser scanning microscope (Zeiss LSM700). The TB-LN and BAL fluid were disrupted to generate single-cell suspensions and acquired on a flow cytometer (FACSLyric; BD). Both the supernatant fluid and the BAL cells were used for infectious virus isolation on AO at ALI. All tissues (whole head, septum, concha, TB-LN, trachea, and formalin-inflated lungs) were stored in formalin for histology.

### RNA isolation and RT-qPCR on throat and nose swabs.

From the throat and nose swabs viral RNA was isolated as described previously (59). RNA was directly used for RT-qPCR using primers and probes for HPIV-3 (80).

### IHC and dual indirect immunofluorescence assay of formalin-fixed tissues and cells.

Lungs, concha, and lymph nodes were stored in formalin and directly embedded in paraffin as described previously (67). Whole heads, nasal septa, and trachea were decalcified in 10% (wt/vol) EDTA for at least 4 weeks before paraffin embedding. Thin sections (3 μm) were prepared from the formalin-fixed, paraffin-embedded tissues and stained using hematoxylin and eosin. rHPIV-3 CI-1-EGFP distribution and tropism in the whole heads, nasal septa, concha, TB-LN, and lungs was assessed by immunohistochemistry using a polyclonal rabbit anti-GFP antibody as described previously (67, 81). The human and ferret AO were fixed in 4% paraformaldehyde and stained with antibodies for ZO-1, acetylated α-tubulin, and with Hoechst as described previously (75).

### Detection of HPIV-3-EGFP neutralizing antibodies.

Virus neutralizing antibodies at −4, 0, 7, 14, and 21 dpi were detected by an endpoint titration assay. Triplicates of ferret sera were incubated with 100 TCID_50_ rHPIV-3 CI-1-EGFP in a 2-fold dilution series starting at a concentration of 1:8 for 2 h at 37°C. The virus-serum mixture was added to Vero cells and incubated for 2 to 3 days at 37°C and 5% (vol/vol) CO_2_. Green fluorescence was used as a readout to determine the minimal serum concentration required to inhibit viral replication (59). Additionally, a selection of samples and serum dilutions was overlaid on AO to confirm virus neutralization in primary cells.
